# Seizure outcomes and complications associated with stereoelectroencephalography versus subdural electrodes for invasive monitoring in epilepsy surgery: a meta-analysis

**DOI:** 10.3389/fneur.2025.1619288

**Published:** 2025-10-10

**Authors:** Nallammai Muthiah, Hope M. Reecher, Seyed Farzad Maroufi, Alireza Mansouri, Emily Harford, Taylor J. Abel

**Affiliations:** ^1^Department of Neurological Surgery, Washington University School of Medicine, St. Louis, MO, United States; ^2^Department of Neurological Surgery, Medical College of Wisconsin, Milwaukee, WI, United States; ^3^Department of Neurosurgery, Tehran University of Medical Sciences, Tehran, Iran; ^4^Department of Neurological Surgery, Penn State University, Hershey, PA, United States; ^5^Department of Neurological Surgery, University of Pittsburgh, Pittsburgh, PA, United States

**Keywords:** SEEG, SDE, seizure freedom, complications, invasive monitoring

## Abstract

**Introduction:**

Approximately 1.2% of people in the United States have epilepsy. Accurate identification of seizure origin is critical for clinical management. Yan et al. published a systematic review up to 2018 comparing SDE and SEEG (two invasive monitoring modalities) on clinical effectiveness and safety. However, meta-analysis was not performed, and multiple centers have published key SDE and SEEG data since 2018.

**Methods:**

We performed an updated literature search from Yan et al., through June 2023, of studies on patients who underwent SEEG or SDE for seizure focus localization. Inclusion criteria were: (1) randomized control trial, prospective or retrospective cohort study, or case series >5 patients, (2) at least one patient who underwent seizure focus resection, (3) outcomes of *either* seizure freedom *or* complications. Meta-5analytic methods were utilized for data analysis.

**Results:**

An initial search resulted in 4,647 records; 81 studies were included, reflecting 3,482 SEEG and 2,816 SDE patients. Compared to SEEG, SDE exhibited similar operative time (164 vs. 185 min, *p* = 0.50), inpatient monitoring time (8.7 vs. 8.9 days, *p* = 0.81), and length of hospital stay (11.8 vs. 9.7 days, *p* = 0.17). Seizure foci were identified in 95.4% of SEEG patients and 91.9% of SDE patients (*p* = 0.25). A higher proportion of SDE patients underwent resective surgery (85.6 vs. 74.0%, *p* < 0.01). Overall, 8.0% of SEEG patients and 10.6% of SDE patients experienced adverse events (*p* = 0.22). Incidence of infection was higher for SDE (1.8%) than for SEEG (0.3%, *p* < 0.01). Overall, 62.7% of SEEG patients and 63.4% of SDE patients achieved seizure freedom (*p* = 0.87). Among studies which directly compared SEEG to SDE, there were no differences in seizure freedom attainment or overall morbidity.

**Conclusion:**

SEEG and SDE are safe and effective modalities to localize seizure foci. SDE was associated with higher rates of subsequent resection, but infection rate was higher for SDE than SEEG.

## 1 Introduction

Approximately 1.2% of people in the United States have epilepsy, which reflects a population of 3.4 million nationwide (1). Approximately 30% of patients with epilepsy will have seizures resistant to antiseizure medications (ASMs) (2). In focal epilepsy, surgical resection is associated with decreased seizure frequency (3–6), increased rate of seizure freedom (6), improved quality of life (7), and better behavioral outcomes compared to ASM therapy alone. Accurately identifying the epileptogenic zone (EZ) for surgical resection is crucial. Scalp electroencephalography (EEG), magnetic resonance imaging (MRI), and semiology analysis are a few common non-invasive methods to identify the EZ. For up to 50% of resective epilepsy surgeries, invasive monitoring with direct brain recordings is necessary to identify the EZ. Invasive monitoring in epilepsy includes both stereoelectroencephalography (SEEG) and subdural electrodes (SDE). While SEEG utilizes thin, depth electrodes to sample superficial and deep cortex, SDE requires a craniotomy and records from the cortical surface of the brain. For the last decade, there has been extensive investigation into the comparative effectiveness of SEEG and SDE regarding (1) clinical effectiveness and (2) safety.

In 2019, Yan et al. published a systematic review comparing SEEG and SDE, reporting that SEEG was associated with higher seizure freedom rates after resection, lower mortality rates, and lower morbidity rates compared to SDE (8). However, this systematic review was limited because at the time, few centers outside of Europe had published their results. Of note, in 2021, Jehi et al. performed a registry analysis of 10 surgical epilepsy centers, reflecting 2,012 patients, to compare rates of surgical resection, seizure freedom, and complications among patients who underwent invasive intracranial monitoring with SDE versus SEEG (7). They found that while surgical resection rate was higher for those who underwent SDE, the complication rate and seizure freedom rates were both lower compared to SEEG (9). Changes in utilization of these invasive monitoring methods may affect clinical outcomes, given increased collective institutional experience. Therefore, the general objectives of this study were to (1) update the systematic review performed by Yan et al. in 2019, (2) to rigorously analyze SEEG and SDE seizure freedom outcomes and complication rates using meta-analytic methods, and (3) incorporate individual analyses into the meta-analysis that directly compared SDE and SEEG. We hypothesized that, akin to what Jehi et al. demonstrated in 2021, SEEG would be associated with a higher seizure freedom rate and a lower complication rate than SDE.

## 2 Methods

This study was registered on the PROSPERO database and was performed following Preferred Reporting Items of Systematic Reviews and Meta-Analyses (PRISMA) guidelines.

### 2.1 Information sources and search strategy

We performed an updated literature search relative to Yan et al.’s November 2018 literature search of the Embase, Cochrane, and Ovid MEDLINE databases in December 2021 with the assistance of a librarian (M.A.). Appropriate keywords and subject headings for each database were used for the two concepts, epilepsy (epilepsy, seizure, etc.) and SEEG (stereoelectroencephalography, brain mapping, etc.). Results were limited to primary research articles about humans >2 years of age, written in English, and published since 1946. Conference materials were removed from Embase results. The literature search was subsequently updated in June 2023 to identify any additional articles for inclusion.

The reference lists of retrieved articles were screened by abstract for content and relevance to the research question to identify relevant articles for further review. Only studies written in English were included. Two investigators (N.M. and H.M.R) independently conducted the literature search, screen of abstracts, and selection of included trials.

### 2.2 Study selection

The goal of the selection process was to review and add to the prior systematic literature search performed by Yan et al. in 2018. Thus, our study selection criteria were identical. We included studies of patients with ASM-resistant epilepsy who underwent SEEG or SDE for seizure foci localization. Studies were included if they: (1) were a randomized control trial, prospective cohort study, retrospective cohort study, or a case series >5 patients, (2) had at least 1 patient who underwent seizure foci resection, (3) had outcomes of *either* seizure freedom *or* complications. As opposed to Yan et al.’s methodology, studies that reported on both SDE and SEEG outcomes were included in this analysis and were separately analyzed.

Studies with 5 or fewer patients were excluded from analysis. Other exclusion criteria were: (1) no primary data reported, (2) surgical resection was not a treatment that any patient in the study underwent (i.e., all underwent laser ablation, vagus nerve stimulation, or corpus callosotomy, etc.), (3) all patients underwent resective surgery irrespective of SEEG or SDE analysis, (4) overt selection bias (e.g., all patients had temporal lesion, increasing likelihood of resective surgery), and (5) the paper was not in English. Thermocoagulation and laser interstitial thermal therapy (LITT) were excluded, as these alternative treatment techniques have different seizure freedom rates compared to open resection, prohibiting a direct comparison between SEEG and SDE. There were no exclusions based on age. When duplicate studies were found (i.e., studies from the same epilepsy program over the same or overlapping time frames of data reporting), only the study with the least missing data or the most current study was included for quantitative assessment.

### 2.3 Data collection and critical appraisal

Each article was reviewed by two investigators independently (N.M. and H.M.R). A data collection form was utilized to create a record for each study and all data were extracted from article text, tables, and figures. Data extracted from articles included author name; year of publication; baseline characteristics, such as age and sex; epilepsy information, such as duration of epilepsy, etiology of temporal origin, tumor, focal cortical dysplasia type 1 (FCD1), FCD2, or lesional; and seizure outcomes, such as percentage seizure freedom following resective surgery, percentage seizure freedom independent of surgery, Engel class, and International League Against Epilepsy (ILAE) grading. Morbidity and mortality data were collected, with specific numbers related to all hemorrhages, intracranial hemorrhage, subdural or epidural hemorrhage, overall infection, meningitis, abscesses, or superficial hemorrhage, cerebrospinal fluid (CSF) leak, lead fractures, transient neurologic deficit, permanent neurologic deficit, and medical complications. Variables were collected as overall counts of participants, means, medians, and ranges where appropriate and available.

### 2.4 Study risk of bias assessment

The quality of the included studies was evaluated using the Methodological Index for Non-Randomized Studies (MINORS), designed for observational studies. Each study had a risk score for bias ranging from 0 to 16, with ≥13 being low-risk, 12–9 being moderate-risk, and ≤8 being high-risk. Risk of bias was assessed independently by two authors (N.M. and H.M.R).

### 2.5 Statistical analysis

All analyses were conducted in R statistical analysis software (version 4.1.2, R Foundation for Statistical Computing) using the “meta” package. Event outcomes were pooled using the “metaprop” function, which allows for calculation of 95% confidence intervals with the score statistic and the exact binomial method (10). Continuous outcomes were analyzed using the “metamean” function, which allows for comparison of difference of means. Due to inherent heterogeneity associated with surgical treatments and outcomes, a random effect model was used for reporting outputs. To explore differences in pooled outcomes between monitoring approaches, we conducted subgroup analyses with a Chi-squared tests for subgroup differences as implemented in the “meta” package in R. Meta-regression was conducted to assess the impact of a specific covariate on observed effect. A *p*-value less than 0.05 was considered significant.

## 3 Results

**Figure 1:**
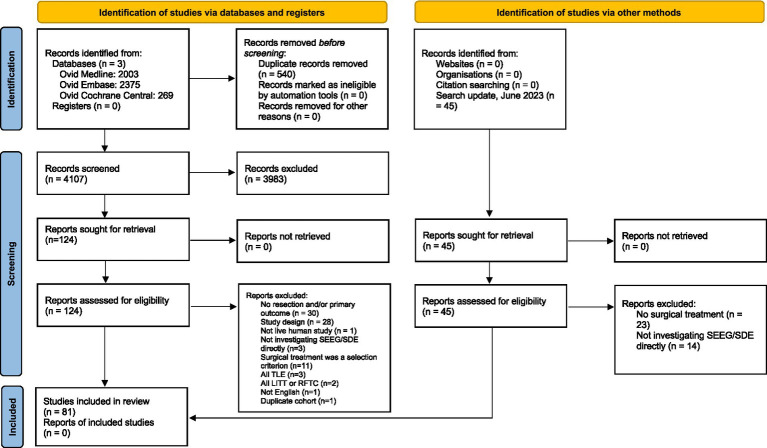
PRISMA flow diagram for study identification, screening, review, and inclusion.

Our search string yielded a total of 4,647 records: 2,003 records from Ovid MEDLINE, 2,375 records from Embase, and 269 records from Cochrane Central. After duplicate removal (*n* = 540), 4,107 records were screened for title and abstract content. After title and abstract screening by our pre-defined inclusion and exclusion criteria, 124 articles were reviewed in detail via full text. Adding the 9 articles identified through the June 2023 search update, a total of 81 studies were included in this meta-analysis. The flow diagram of the screening process is depicted in Figure 1.

The 81 included studies were published between 1990 and 2023. Most were retrospective observational studies (Table 1). The studies were conducted in several countries including the United States, Sweden, and Korea. These included a total of 6,298 patients with drug-resistant epilepsy, 3,482 who were assessed using SEEG and 2,816 patients who underwent SDE. Among these, 24 studies with 1,350 patients (753 SEEG and 597 SDE) reflected only pediatric patients, 11 studies with 290 patients (225 SEEG and 65 SDE) reflected only adult patients, and 41 studies with 3,917 patients reflected both pediatric and adult patients (2,259 SEEG and 1,658 SDE). The remaining 5 studies with 741 patients (245 SEEG and 496 SDE) did not include sufficient detail to determine whether study samples comprised adult and/or pediatric patients.

**Table 1:** Studies included in this meta-analysis, detailed by authors, location of study, methodology of study, sample size, patient demographics, and seizure freedom/morbidity outcomes.

Reference	Method of invasive EEG	Country	Design	Patients, n	Average age (years, range)	Male, %	Postresection seizure freedom, %	Morbidity, %
Van Veelen et al. (17)	SDE	Utrecht, Netherlands	R CS	28	NR	NR	62	NR
Adelson et al. (18)	SDE	Boston, USA	R CS	31	1–18	41.9	NR	16.1
Behrens et al. (19)	SDE	Bonn, Germany	R CS	160	3–55	48.8	NR	3.8
Swartz et al. (20)	SDE	Los Angeles, USA	P CS	55	NR	NR	47	22
Wiggins et al. (21)	SDE	Detroit, USA	R CS	38	18–70	52.6	48.5	24
Asano et al. (22)	SDE	Detroit, USA	R CS	13	1.2–15.4	53.9	84.6	NR
Onal et al. (23)	SDE	Ontario, Canada	R CS	35	2–19	48.6	42.4	57.1
Johnston et al. (24)	SDE	St. Louis, USA	R CS	112	0.83–21.7	47.3	NR	26
Fountas & Smith (25)	SDE	Augusta, USA	R CS	185	16.49	56.2	67.8	8.1
Van Gompel et al. (26)	SDE	Rochester, USA	R CS	198	1–73	52.5	47	14
Wong et al. (27)	SDE	New South Wales, Australia	R CS	71	NR	62	63.8	26.7
Liubinas et al. (28)	SDE	Victoria, Australia	R CS	8	21–47	2.5	87.5	25
Placantonakis et al. (29)	SDE	New York, USA	R CS	26	17–60	65.4	70.6	7.7
Ramananti et al. (30)	SDE	Freiburg, Germany	R CS	14	14–50	35.7	50	NR
Bekelis et al. (31)	SDE	Lebanon, USA	R CS	50	11–58	64	71	6
Vale et al. (32)	SDE	Tampa, USA	R CS	91	11–60	60.4	34.3	11
Abuelem et al. (33)	SDE	Houston, USA	R CS	24	2.5–47	66.7	56	4.2
Hedegard et al. (34)	SDE	Sweden	P CS	276	2–58	51.4	NR	4.8
Kanazawa et al. (35)	SDE	Kyoto, Japan	R CS	16	NR	62.5	87.5	NR
Yang et al. (36)	SDE	Fuzhou, China	R CS	137	NR	61.3	48.9	21.1
Raftopoulos et al. (37)	SDE	Brussels, Belgium	R CS	38	0.4–54	50	NR	2.6
Sakuraba et al. (38)	SDE	Sendai, Japan	R CS	13	12–41	38.5	50	NR
Delev et al. (39)	SDE	Bonn, Germany	R CS	100	2–60	58	53.5	6
Mullin et al. (40)	SDE	Cleveland, USA	R CS	102	5–64	48	70	NR
Hill et al. (41)	SDE	New York, USA	R CS	199	4.7–55.3	49.8	47.1	5.8
Meng et al. (42)	SDE	Ontario, Canada	R CS	199	1.5–18	NR	NR	7.1
Nagahama et al. (43)	SDE	Iowa City, USA	R CS	91	3–62	53.8	65.3	8.8
Schneider et al. (44)	SDE	Berlin, Germany	R CS	58	NR	50	84	7
Kim et al. (45)	SDE & SEEG	Stanford, USA	R CS	22 SEEG 16 SDE	NR	54.6 SEEG 75 SDE	73.3 SEEG 78.6 SDE	0 SEEG 0 SDE
Joswig et al. (46)	SDE & SEEG	London, Canada	P CS & R CS	145 SEEG 355 SDE	NR	49 SEEG 50.1 SDE	NR	2.8 SEEG NR SDE
Kim et al. (47)	SDE & SEEG	Stanford, USA	R CS	47 SEEG 19 SDE	NR	59.6 SEEG 61.1 SDE	29.2 SEEG 35.2 SDE	14.9 SEEG 10.5 SDE
Munari et al. (48)	SEEG	Grenoble, France	R CS	70	NR	36	NR	10
Guenot et al. (49)	SEEG	Lyon, France	R CS	100	NR	45	NR	5
Aghakhani et al. (50)	SEEG	Québec, Canada	R CS	8	16–43	62.5	33.3	12.5
Kassiri et al. (51)	SEEG	Alberta, Canada	R CS	18	3–18	63.6	100	5.6
Serletis et al. (52)	SEEG	Little Rock, USA	P CS	200	3–69	47	67.8	4.5
Taussig et al. (53)	SEEG	Paris, France	R CS	65	1.7–17.1	NR	67	0
Munyon et al. (54)	SEEG	Cleveland, USA	R CS	20	8–59	45	NR	10
Surseh et al. (55)	SEEG	Cleveland, USA	R CS	18	23–56	55.6	76	NR
Roessler et al. (56)	SEEG	Erlangen, Germany	R CS	6	20–37	50	50	0
Li et al. (57)	SEEG	Shanghai, China	R CS	7	5.3–34	57.1	85.7	NR
Lamarche et al. (58)	SEEG	Grenoble, France	R CS	37	6–52	48.6	55	NR
Bourdillon et al. (59)	SEEG	Lyon, France	P CS	525	NR	NR	NR	3.6
Minkin et al. (60)	SEEG	Sofia, Bulgaria	R CS	34	4–58	55.9	NR	0
Ollivier et al. (61)	SEEG	Strasbourg, France	P CS	66	2–53	62.1	NR	15.2
Delev et al. (62)	SEEG	Bonn, Germany	R CS	14	8–47	64.3	61.5	0
Budke et al. (63)	SEEG	Madrid, Spain	R CS	15	3–16	53.5	NR	6.7
Salado et al. (64)	SEEG	Nancy, France	R CS	99	8–53	55.6	NR	12
Dewan et al. (65)	SEEG	Nashville, USA	R CS	15	9–65	73.3	NR	26.7
Abel 2018 (66)	SEEG	Ontario, Canada	R CS	17	3–18	NR	28.6	35
Goldstein et al. (67)	SEEG	New York, USA	R CS	25	5–21	40	53.5	12
Lagarde et al. (68)	SEEG	Marseille, France	R CS	59	2.8–56	51	57.6	NR
Carlson et al. (69)	SEEG	Los Angeles, USA	R CS	52	16–70	50	NR	23
Candela-Canto et al. (70)	SEEG	Barcelona, Spain	P CS	14	5–18	NR	NR	14.2
McGovern et al. (71)	SEEG	Cleveland, USA	R CS	57	NR	56.1	50	9.4
Thorsteinsdottir et al. (72)	SEEG	Munich, Germany	P CS	85	2.5–59.7	48.6	83	5.9
Cardinale et al. (73)	SEEG	Milan, Italy	R CS	713	2–56	56.1	NR	1.8
D’Agostino et al. (74)	SEEG	Lebanon, USA	R CS	13	21–57	77	100	30.8
Peedicail et al. (75)	SEEG	Calgary, Canada	R CS	69	18–70	55.1	26.8	15
Kappen et al. (76)	SEEG	London, UK	R CS	29	1–20	44.8	NR	5
Chaitanya et al. (77)	SEEG	Birmingham, USA	P CS	24	23–61	45.8	NR	20.8
Mereaux et al. (78)	SEEG	Normandy, France	R CS	46	NR	45.7	65.2	10.8
Nelson et al. (79)	SEEG	Washington DC, USA	R CS	19	2–21	74	21	0
Liu et al. (80)	SEEG	Beijing, China	R CS	72	1.8–18	72.2	64.2	5.6
Urgun et al. (81)	SEEG	Orange, USA	R CS	7	22–52	85.7	NR	0
Zheng et al. (82)	SEEG	Shijiazhuang, China	R CS	33	NR	48.5	75	18.2
Durica et al. (83)	SEEG	Dallas, USA	R CS	9	20–37	55.5	50	0
Tsuboyama et al. (84)	SEEG	Boston, USA	R CS	104	NR	54.8	65.7	0
Mavridis et al. (85)	SEEG	Birmingham, UK	R CS	63	4–18	39.1	60.8	6.3
Vakharia et al. (86)	SEEG	London UK	RCT	32	21.2–47.5	56.3	NR	9.4
Hyslop et al. (87)	SEEG	Miami, USA	R CS	44	NR	38.6	41.4	6.8
Belohlavkova et al. (88)	SEEG	Prague, Czech Republic	R CS	19	2.9–18.6	57.9	81.6	31.6
Toledano et al. (89)	SEEG	Madrid, Spain	R CS	71	4–59	62.0	58.2	4.2
Remick et al. (90)	SDE & SEEG	Pittsburgh, USA	R CS	134 SEEG 42 SDE	NR	57.1 SEEG 55.2 SDE	60.2 SEEG 75 SDE	7.1 SEEG 17.9 SDE
Stone et al. (91)	SEEG	Boston, USA	R CS	20	4–23	50	47.4	NR
Larrew et al. (92)	SEEG	Cincinnati, USA	R CS	29	NR	51.7	NR	10.3
Zhao et al. (93)	SEEG	Beijing, China	R CS	32	7–49	46.9	57.1	NR
Mawsawa et al. (94)	SDE & SEEG	Nagoya, Aichi, Japan	R CS	12 SEEG 4 SDE	9–58	NR	NR	0 SEEG 25 SDE
Rijal et al. (95)	SDE & SEEG	Boston, USA	R CS	7 SEEG 12 SDE	4–18 SEEG 2.2–22 SDE	28.6 SEEG 66.7 SDE	100 SEEG 66.7 SDE	NR
Santalucia et al. (96)	SEEG	Brussels, Belgium	R CS	29	3–54	48.3	75	NR
Smith et al. (97)	SEEG	Rochester, USA	R CS	12	12–66	66.7	33.3	NR

Studies included in this meta-analysis, detailed by authors, location of study, methodology of study, sample size, patient demographics, and seizure freedom/morbidity outcomes. SDE: subdural electrodes, SEEG: stereoelectroencephalography, P CS: prospective case series/cohort study, R CS: retrospective case series/cohort study, RCT: randomized control trial, NR: not reported.

### 3.1 Baseline cohort characteristics

Table 2 demonstrates baseline cohort characteristics for SEEG and SDE patients in this sample. The mean age of patients who underwent SEEG ranged from 8.2 to 40 years with a pooled mean of 23.0 years, while the mean age in the SDE group ranged from 6.7 to 37.7 years with a pooled mean of 22.7 years. Within this cohort, 52.4% of SEEG patients and 52.7% of SDE patients were male. The mean age at seizure onset for SEEG patients was 11.0 years, while it was 7.0 years for SDE patients. The mean duration of epilepsy symptoms was 163.9 months among SEEG patients and 137.2 months among SDE patients. Etiologies of epilepsy were distributed similarly among the two cohorts, with 35% (SEEG) and 30% (SDE) of epilepsy being temporal lobe epilepsy and 57% (SEEG) and 59% (SDE) being lesion-related epilepsy (i.e., tumor, tuber, focal cortical dysplasia, etc.). No baseline characteristics differed significantly between the two invasive monitoring techniques.

**Table 2:** Summary of weighted mean and weighted percent comparisons between SEEG and SDE cohorts.

Variable	SEEG (51 studies, 3,279 patients)	SDE (36 studies, 2,995 patients)	*p* value
Age (y), mean Weighted Mean [95% CI]	47 studies, 2,602 patients 23.03 [20.18 to 25.88]	32 studies, 2,852 patients 22.73 [19.75 to 25.70]	0.89
Male, % Weighted % [95% CI]	44 studies, 2,648 patients 52.43% [49.47–55.38%]	30 studies, 2,519 patients 52.75% [49.87–55.61%]	0.88
Age at Onset (y) Weighted Mean [95% CI]	21 studies, 715 patients 10.96 [8.44–13.48]	5 studies, 399 patients 6.98 [3.08–10.88]	0.09
Duration of Disease (mo) Weighted Mean [95% CI]	22 studies, 1,025 patients 163.94 [137.77–190.12]	7 studies, 808 patients 137.24 [94.13–180.35]	0.30
Temporal Lobe, % Weighted % [95% CI]	32 studies, 1,299 patients 35.31% [24.96–47.25%]	21 studies, 993 patients 30.48% [20.17–43.2%1]	0.56
Epilepsy etiology			
Brain Tumor, % Weighted % [95% CI]	18 studies, 909 patients 5.96% [3.53–9.89%]	14 studies, 1,220 patients 7.23% [5.18–10.00%]	0.53
FCD 1, % Weighted % [95% CI]	15 studies, 698 patients 15.95% [8.20–28.72%]	6 studies, 611 patients 24.24% [10.59–46.36%]	0.67
FCD 2, % Weighted % [95% CI]	15 studies, 698 patients 10.38% [4.39–22.64%]	6 studies, 611 patients 0.20% [0.00–16.56%]	0.09
FCD 1&2, % Weighted % [95% CI]	27 studies, 1,295 patients 33.96% [26.05–42.89%]	16 studies, 1,164 patients 32.85% [22.04–45.85%]	0.88
Any lesional Epilepsy, % Weighted % [95% CI]	36 studies, 1,596 patients 57.42% [48.74–65.67%]	19 studies, 1,234 patients 59.39% [46.16–71.38%]	0.80
Tuberous Sclerosis, % Weighted % [95% CI]	10 studies, 308 patients 3.70% [1.46–9.05%]	9 studies, 385 patients 7.62% [1.25–34.94%]	0.48

Summary of weighted mean and weighted percent comparisons between SEEG and SDE cohorts. SEEG: stereotactic electroencephalography, SDE: subdural electrodes, FCD: Focal cortical dysplasia, CI: confidence interval.

### 3.2 Intracranial monitoring

Table 3 demonstrates intraoperative variables associated with electrode placement. The mean operative time for electrode placement was 164 and 185 min for the SEEG and SDE groups, respectively (*p* = 0.50). Similarly, inpatient monitoring time was comparable between the SEEG and SDE groups (8.7 vs. 8.9 days, *p* = 0.81). The mean length of hospital stay after electrode placement, while slightly longer in the SDE group, did not significantly differ between the techniques (11.8 vs. 9.7 days, *p* = 0.17). Notably, the “length of hospital stay” for SDE patients included both invasive monitoring duration and surgical resection at the time of electrode removal if it occurred.

**Table 3:** Analysis of intra- and post-operative complications.

Variable	SEEG (51 studies, 3,279 patients)	SDE (36 studies, 2,995 patients)	*p* value
Operation Time (min), mean	15 studies, 508 patients	6 studies, 676 patients	0.5
Weighted Mean [95% CI]	163.66 [136.99–190.33]	185.34 [128.78–241.91]	
Implant Time (days), mean	18 studies, 851 patients	25 studies, 2,122 patients	0.81
Weighted Mean [95% CI]	8.65 [7.44–9.87]	8.85 [7.79–9.91]	
LOS (days), mean	5 studies, 197 patients	4 studies, 393 patients	0.17
Weighted Mean [95% CI]	9.70 [6.76–12.64]	11.80 [11.21–12.38]	
Complications, overall n studies, n patients	41 studies, 2,952 patients	30 studies, 2,785 patients	0.22
Overall Weighted % [95% CI]	8.04 [5.84–10.98]	10.58 [7.79–14.21]	
ICH, Weighted % [95% CI]	3.88 [2.48–6.01]	2.53 [1.55–4.09]	0.2
SDH/EDH, Weighted % [95% CI]	0.89 [0.43–1.84]	1.48 [0.80–2.72]	0.29
Infection, all, Weighted % [95% CI]	0.34 [0.12–0.98]	1.77 [1.08–2.88]	<0.01
Meningitis, Weighted % [95% CI]	0.07 [0.02–0.27]	0.03 [0.00–0.96]	0.66
Abscess, Weighted % [95% CI]	0.07 [0.02–0.27]	0.21 [0.05–0.83]	0.25
Osteomyelitis, Weighted % [95% CI]	0.00 [0.00–1.00]	0.19 [0.04–0.08]	0.99
Superficial Inf, Weighted % [95% CI]	0.16 [0.03–0.74]	0.60 [0.24–1.51]	0.15
CSF Leak, Weighted % [95% CI]	0.00 [0.00–1.00]	0.08 [0.01–0.88]	0.91
Electrode Fracture, Weighted % [95% CI]	0.46 [0.18–1.21]	0.03 [0.00–0.98]	0.14
Neurologic Deficit, total, Weighted % [95% CI]	0.90 [0.41–1.93]	0.75 [0.30–1.83]	0.76
Transient	0.61 [0.24–1.53]	0.55 [0.19–1.55]	0.87
Permanent	0.19 [0.04–0.80]	0.09 [0.01–0.76]	0.55
Mortality	39 studies, 2,928 patients	27 studies, 2,733 patients	0.34
Weighted % [95% CI]	0.20 [0.09–0.46]	0.01 [0.00–4.91]	
Zone Identification	25 studies, 1,213 patients	16 studies, 986 patients	0.25
Weighted % [95% CI]	95.37 [90.72–97.75]	91.92 [85.16–95.76]	
Subsequent Resection	45 studies, 2,644 patients	33 studies, 2,241 patients	**<0.01**
Weighted % [95% CI]	73.95 [65.95–80.62]	85.62 [80.74–89.43]	

Analysis of intra- and post-operative complications. SEEG: stereotactic electroencephalography, SDE: subdural electrodes, LOS: length of stay, SDH: subdural hematoma, EDH: epidural hematoma, ICH: intracerebral hemorrhage, CSF: cerebrospinal fluid, CI: confidence interval.

Table 3 additionally summarizes the morbidity and mortality of SEEG versus SDE. Overall, 8.0% of patients who underwent SEEG and 10.6% of patients who underwent SDE experienced adverse events (*p* = 0.22), with intracranial hemorrhage being the most common adverse event. The pooled mortality of SEEG and SDE were 0.2 and 0.01%, respectively (*p* = 0.34). Invasive monitoring resulted in seizure foci identification in 95.4% of SEEG patients and 91.9% of SDE patients (*p* = 0.25). A higher percentage of SDE patients underwent subsequent resective surgery compared to SEEG (85.6 vs. 74.0%, *p* < 0.01).

### 3.3 Post-monitoring course

Details of follow duration and seizure outcomes are summarized in Table 4. The mean duration of postoperative follow-up was significantly higher in the SDE group (40.8 months) compared to the SEEG group (28.4 months, *p* = 0.01). Most studies which reported seizure freedom outcomes utilized the Engel classification (*n* = 16 SEEG studies reflecting 761 patients and *n* = 6 SDE studies reflecting 164 patients), while a minority utilized the ILAE classification (*n* = 5 SEEG studies reflecting 153 patients and *n* = 2 SDE studies reflecting 106 patients). Of note, some studies which reported Engel classes did not specifically sub-classify the Engel 1 class. Overall, there was no significant difference in seizure freedom outcomes (either ILAE 1 or Engel 1a, both corresponding to complete and sustained seizure freedom), with 62.7% of SEEG patients and 63.4% of SDE patients achieving seizure freedom (*p* = 0.87). Among studies reporting Engel classification, Engel 1a was achieved in 62.7% of SEEG surgeries and 44.9% of SDE surgeries (*p* = 0.08). Among studies reporting ILAE classification, ILAE 1 was achieved in 64.7% of SEEG patients and 53.8% of SDE patients (*p* = 0.08). The distribution of outcomes (either Engel classes or ILAE grades) was similar between the two approaches (*p* = 0.28 for Engel classes and *p* = 0.06 for ILAE grades).

**Table 4:** Weighted mean and percent seizure outcomes per SDE and SEEG studies.

Outcome variable	sEEG (51 studies, 3,279 patients)	SDE (36 studies, 2,995 patients)	p value
Follow-up (mo), mean	24 studies, 879 patients	21 studies, 1,440 patients	**0.01**
Weighted Mean [95% CI]	28.36 [24.52–32.20]	40.78 [31.57–50.00]	
Seizure-Free, %	33 studies, 1,332 patients	28 studies, 1,277 patients	0.87
Weighted % [95% CI]	62.72 [57.90–67.30]	63.39 [56.80–69.52]	
Engel Ia, %	16 studies, 761patients	6 studies, 194 patients	0.08
Weighted % [95% CI]	54.51 [45.87–62.89]	44.85 [37.99–51.90]	
ILAE I, %	5 studies, 153 patients	2 studies, 106 patients	0.08
Weighted % [95% CI]	64.71 [56.82–71.86]	53.77 [4426–63.02]	
Engel Classes	21 studies, 571 patients	18 studies, 875 patients	0.28
I, *n* patients (%)	355 (62.17)	505 (57.71)	
II, *n* patients (%)	92 (16.11)	178 (20.34)	
III, *n* patients (%)	69 (12.08)	117 (13.37)	
IV, *n* patients (%)	55 (9.63)	75 (8.57)	
ILAE Grade	4 studies, 121 patients	2 studies, 106 patients	0.06
I, *n* patients (%)	79 (65.29)	57 (53.77)	
II, *n* patients (%)	10 (8.26)	5 (4.72)	
III, *n* patients (%)	13 (10.74)	9 (8.49)	
IV, *n* patients (%)	12 (9.92)	26 (24.53)	
V, *n* patients (%)	6 (4.96)	8 (7.55)	
VI, *n* patients (%)	1 (0.83)	1 (0.94)	

Weighted mean and percent seizure outcomes per SDE and SEEG studies. SEEG: stereotactic electroencephalography, SDE: subdural electrodes, ILAE: International League Against Epilepsy, CI: confidence interval.

**Figure 2:**
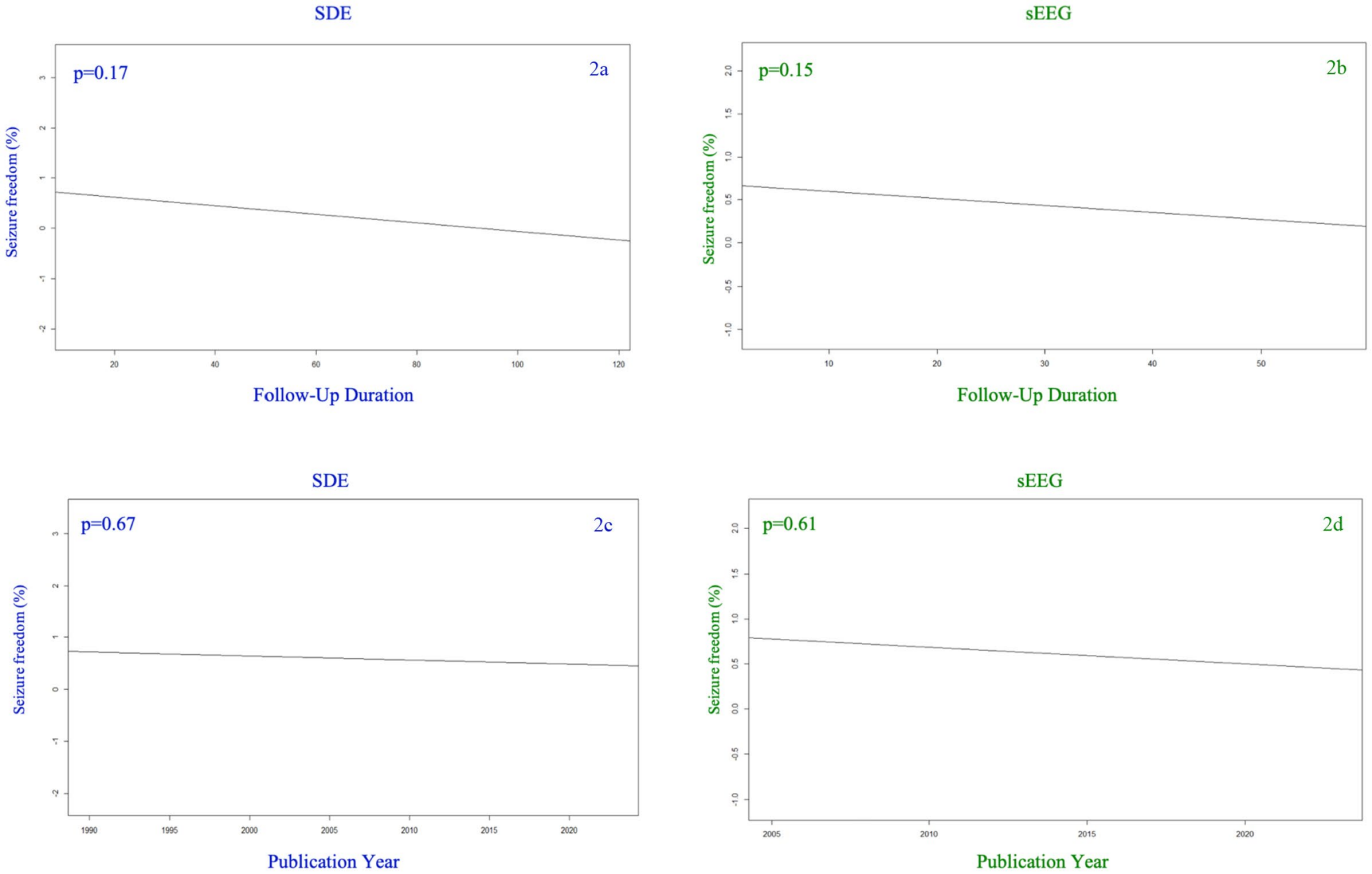
Seizure freedom percentage among SDE (left side) versus sEEG (right side) studies based on the year of publication (top row) and the duration of follow up (months) for the studies reported (bottom row).

Figure 2 demonstrates the rate of seizure freedom as a function of follow-up duration (2a,b) and study publication year (2c,d). This meta-regression demonstrated that the seizure freedom rate was not significantly associated with follow up duration for either cohort (Figures 2a,b, SEEG *p* = 0.15, SDE *p* = 0.10). There was also no significant association between year of study publication and seizure freedom rate for either cohort (Figures 2c,d, SEEG *p* = 0.61, SDE *p* = 0.67).

**Figure 3:**
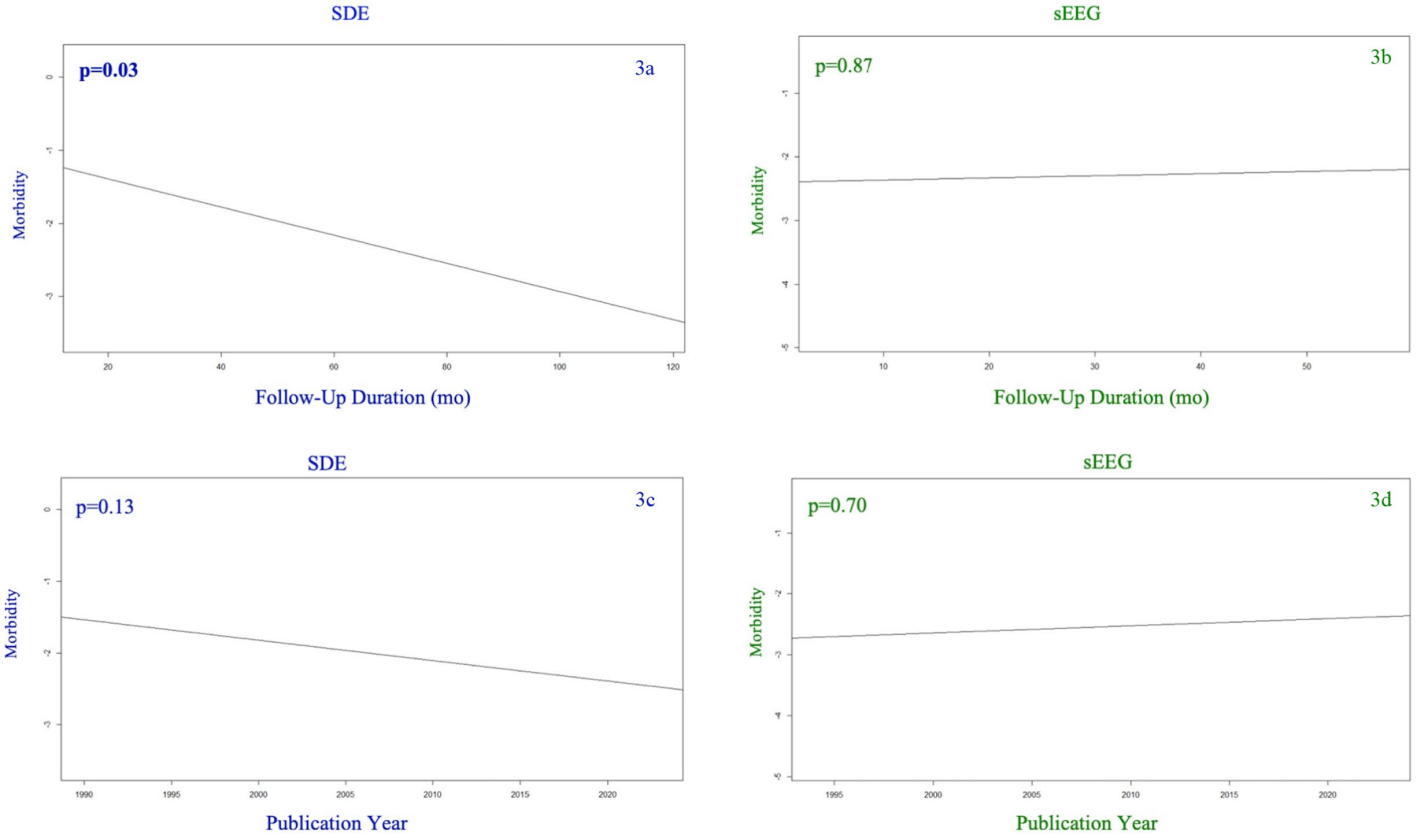
Morbidity (any complication) among SDE (left side) versus sEEG (right side) studies based on the year of publication (top row) and the duration of follow up (months) for the studies reported (bottom row).

Figure 3 demonstrates morbidity as a function of follow-up duration (3a,b) and study publication year (3c,d). While the SEEG group’s overall morbidity rate was affected by neither study publication year nor follow-up duration, the SDE group demonstrated a lower morbidity rate as follow-up duration increased (*p* = 0.03). There was no significant association between publication year and morbidity rate among the SDE group.

### 3.4 Double arm studies

**Figure 4:**
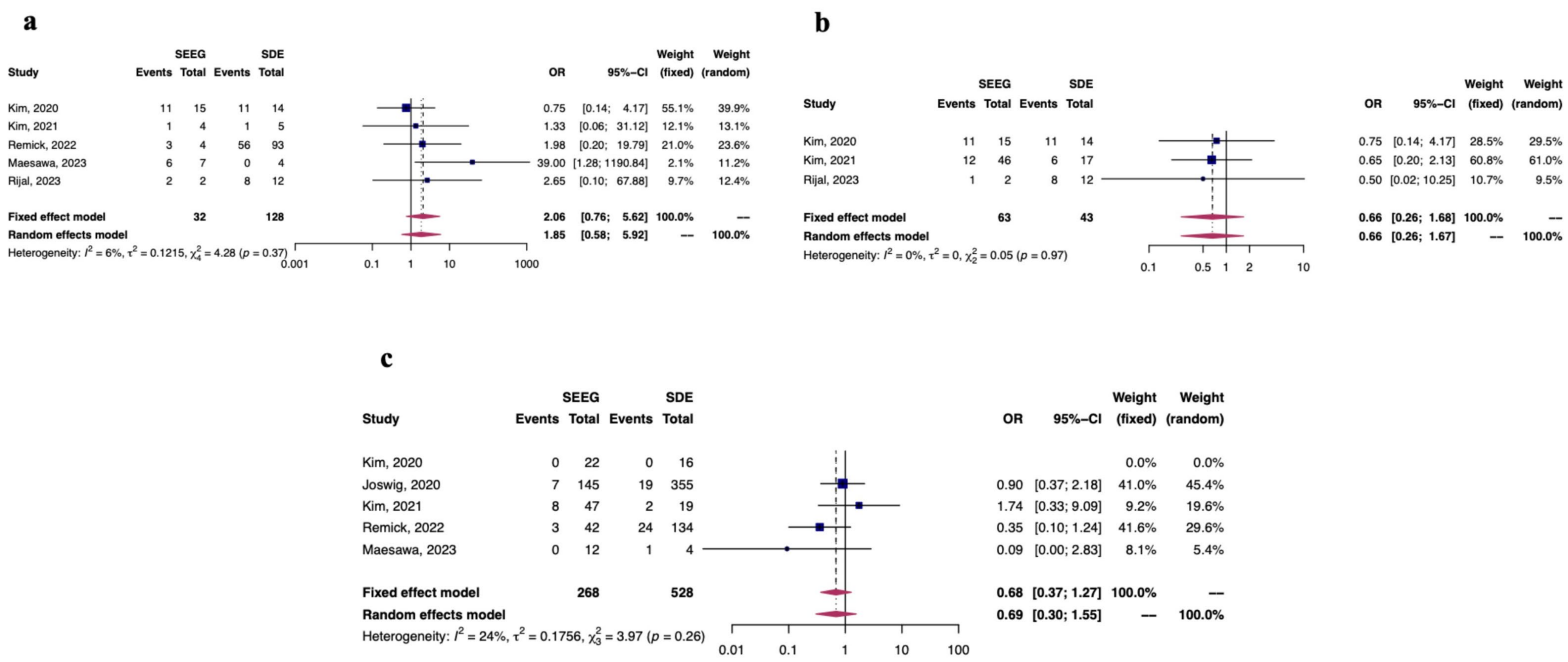
Among studies which directly compared SDE to sEEG (*n* = 6), fixed and random effects model results for seizure freedom **(a)**, Engel 1a outcomes **(b)**, and morbidity outcomes **(c)**.

There were 6 studies in this analysis which reported data on both SEEG and SDE, and 3 reported seizure freedom outcomes using the Engel classification system. Figures 4a–c demonstrate forest plots of seizure freedom between SEEG and SDE (4a), outcome of Engel 1a. between SEEG and SDE (4b), and overall morbidity rates from the invasive monitoring procedure (4c). Overall, there were no differences between SEEG and SDE (among these studies in which the procedures were directly compared) with regards to seizure freedom attainment or overall morbidity.

### 3.5 Risk of bias

The majority of studies included in this analysis (58.0%) demonstrated moderate risk of bias per the MINORS scale, and 25.9% of included studies demonstrated high risk of bias. The primary source of bias was rooted in retrospective study design. Loss to follow up was another source of bias.

## 4 Discussion

In this study, we present the first meta-analysis to directly compare SEEG with SDE, specifically including studies that compared SEEG to SDE directly. Our analysis utilized data from 81 articles, representing 6,274 patients with drug-resistant epilepsy who underwent either SEEG or SDE for seizure foci localization. We found SDE to be associated with significantly increased rate of subsequent surgical resection, but not associated with seizure freedom or morbidity. Further, we found that SDE was associated with an increased infection rate compared to SEEG.

SDE and SEEG are both important, albeit different, surgical techniques to more accurately localize seizure foci. Both techniques involve temporary implantation of electrodes and subsequent inpatient EEG monitoring while electrodes remain in place. In North America, SDE was previously the standard method for invasive EEG recording. SDE typically requires a large craniotomy and only allows for placement of electrodes on the surface of the cortex. In this way, a large portion of the cortical surface can be covered due to the size of the electrode grids and strips as well as the large craniotomy required for implantation. A relative advantage of SDE is this broad cortical surface coverage, which allows for easier implementation of functional mapping compared to SEEG (97). However, not all seizures originate from the cortical surface. For patients with seizure foci deeper than can be detected by subdural electrodes, SDE may not adequately capture the EZ with SDE unless depth electrodes are also implanted. In the past decade, SEEG techniques have become rapidly adopted across North America. SEEG is a less invasive technique which involves implanting depth electrodes through a percutaneous stereotactic approach. SEEG offers the unique advantages of avoiding a large craniotomy, implantation in both hemispheres when necessary, and access to deep cortical structures. Furthermore, with the increased availability of surgical robots such as ROSA, SEEG electrode implantation is more accurate, efficient, and safe. The startup of an SEEG program can be somewhat burdensome to institutions, however, as equipment is costly and requires specialized training (98).

Our analysis revealed no differences in surgical electrode placement time (185 min for SDE versus 163 min for SEEG), monitoring time (8.9 days for SDE vs. 8.6 days for SEEG), or length of hospital stay (11.8 days for SDE vs. 9.7 days for SEEG) between procedures. Recent evidence had demonstrated that with the advancements in neurosurgical robotics, SEEG cases were approximately one-third the duration of SDE cases (11). However, in our analysis, we did not specifically ascertain which SEEG procedures were performed with robots versus without, nor did we obtain the size of the craniotomies performed for SDE procedures. Thus, there is heterogeneity in the data, which may explain the lack of difference between procedures. Additionally, as mentioned earlier, experience has potentially optimized both surgeons’ efficiency with their preferred procedure and subsequent postoperative patient care.

SEEG successfully localized 95% of seizure foci, while SDE localized 92% of seizure foci. Although this was not statistically significant, it is a clinically important finding that a similar proportion of patients can achieve seizure foci localization with a less invasive procedure. Interestingly, a higher proportion of SDE patients underwent surgical resection of seizure than SEEG patients. This may be secondary to the fact that SDE involves a craniotomy for electrode implantation and requires reopening of the craniotomy for explantation. Therefore, if seizure foci are identified, they are easily accessible at the time of SDE explantation. For SEEG patients, seizure foci resection does not necessarily need to involve open craniotomy. Patients may opt to continue with minimally invasive approaches for treatment if the location is amenable, including LITT or radiofrequency ablation, and these patients are not reflected in our analysis. Further, localization by SEEG may afford the opportunity to resect seizure foci through a smaller incision than used to implant SDE electrodes.

We also found no difference in subsequent resection or seizure freedom rates between SEEG and SDE. This contrasts with Yan et al.’s previous finding (8), though it substantiates early evidence that the two procedures may be similar in effectiveness. The reason for this finding is likely multifaceted. As institutions have gained more experience with SEEG or SDE over the last several years, it is feasible that centers have opted primarily for one over the other. Therefore, each institution has developed expertise in their preferred method of invasive EEG. It is feasible that with this increased experience, surgeons have improved in the following aspects: selecting appropriate patients for each procedure, identifying cortical areas of interest based on non-invasive diagnostic studies, performing surgical implantation safely and precisely, interpreting data with more nuance, and/or improved management of patients postoperatively. In short, perhaps institutions are becoming better experts in their chosen invasive EEG method.

We were curious as to whether a study’s publication year or reported follow-up duration were associated with the finding that seizure freedom was similar between SEEG and SDE. As our baseline analysis demonstrated, studies in the SDE cohort reported longer follow up duration than studies in the SEEG cohort. This is likely secondary to the fact that SEEG is a more newly adopted technique, which is substantiated by the finding that studies published in 2018 or later were significantly biased toward reporting on SEEG (*p* < 0.001; Chi-squared test). Despite this, we found no significant association between either year of publication or follow-up duration and seizure freedom in either of our cohorts. We conducted a similar analysis for morbidity and found the SDE group demonstrated a lower morbidity rate as follow-up duration increased (*p* = 0.03). This is an interesting finding though it is without a clear explanation.

Some centers have rapidly adopted SEEG and have shown excellent results with the technique. Indeed, a recent large-scale, longitudinal, single-center analysis by Tandon et al. demonstrated that the adoption of SEEG was associated with lower complication rates, less narcotic utilization, and improved seizure localization compared to SDE (9). Lower resection rates following SEEG compared to SDE have been demonstrated previously (11). In being more selective with offering surgical resection, only patients with the greatest likelihood of having a positive outcome undergo the procedure. This minimizes the proportion of patients who undergo surgical resection and continue to have seizures. However, our finding may be interpreted from another perspective. It is possible that while SEEG was successful at localizing 95% of seizure foci, these foci were not able to be feasibly resected, which may have negatively influenced the seizure freedom rate. SEEG has been utilized for complex, difficult-to-localize seizure foci (12), which may influence outcomes following this procedure. Additionally, SEEG may be a preferred option in nonlateralized epilepsy as it avoids the requirement for bilateral craniotomies (97).

Regarding morbidity and mortality, 8.0% of patients who underwent SEEG and 10.6% of those who underwent SDE experienced any postoperative morbidity. The most common complication was intracranial hemorrhage (3.9% among SEEG patients and 2.5% among SDE patients). SDE was associated with a significantly higher infection rate than SEEG (1.8% versus 0.3%, respectively). This is consistent with existing literature. Given that SDEs are associated with larger incisions and larger exposed surface area, infection rates have been demonstrated to be higher for SDE procedures when compared to SEEG (11, 12). The incidence of intracranial hemorrhage between the two procedures is mixed. Even though SDE involves a larger incision and craniotomy, several small burr holes and depth electrodes are typically placed for SEEG, each of which can also be associated with small subdural and epidural hemorrhages as well as tract hemorrhages or small intraparenchymal hemorrhages along the electrode trajectory. Most studies did not report on the size or clinical significance of these intracranial hemorrhages (i.e., if they were able to be followed with serial imaging or required reoperation), so this nuance is missing in our meta-analysis. There were similar rates of CSF leak, electrode fracture, and neurologic deficits (both temporary and permanent) between groups. Finally, we found there was no difference in the mortality rate between SDE and SEEG (0.01 vs. 0.2%, respectively).

### 4.1 Limitations

This study has several limitations which are important to discuss. Firstly, it is important to note that there is likely strong selection bias as to which patients undergo SDE versus SEEG. Because of the requirement of craniotomy for SDE, often at institutions where SDE is common, only patients who are expected to eventually need a craniotomy for surgical resection based on pre-invasive monitoring data undergo SDE monitoring. In other words, while SEEG is more broadly utilized in epilepsy patients, SDE may be implanted in patients with a higher pre-test probability for resection. Furthermore, studies included in this analysis, while rigorous, were heterogenous. There are several confounders for which we are not able to account and may affect the primary outcomes of this analysis. Not all studies included potentially useful demographic data. For example, not all studies mentioned whether patients had clear lesions on MRI, if epilepsy was associated with prior traumatic brain injury, or if epilepsy was thought to be congenital. Still, we rigorously excluded studies with clear biases that could influence the decision to pursue surgical resection. For example, we excluded studies which reported exclusively temporal lobe epilepsy patients. An important avenue for subsequent research is to segregate etiologies of epilepsy and assess if SEEG or SDE are more likely to allow a certain population to attain seizure freedom. Similar to the Yan et al. study, ours is also limited by the heterogeneity of technique. Subdural electrodes may involve strips, grids, or a combination of both and can be placed via burr holes or craniotomies, while SEEG can be placed with a robot or manually. Further, both techniques can involve implantation with unilateral or bilateral coverage. These variables can significantly affect the operative time as well as the complication rates of these two procedures. Even in the perioperative period, there is heterogeneity in medical management. For example, some institutions give antibiotics for the duration of invasive monitoring, while other dose antibiotics only at the time of implantation, and this may possibly affect infection incidence. Not all manuscripts had the same rigor in reporting methodology, so the collection of data and synthesis of conclusions is limited. Finally, we acknowledge the years in which this meta-analysis includes, ending in 2023, which may exclude new studies that have been published within the last year.

## 5 Conclusion

In this meta-analysis comparing SEEG to SDE, utilizing data from 81 articles, representing 6,298 patients, we demonstrate that SEEG and SDE have similar proportions of patients who subsequently achieve seizure freedom. While SDE patients are more likely to undergo open resective surgery after successful seizure foci localization, SDE was also associated with a higher infection rate than SEEG. The mortality rate of both invasive monitoring procedures is low. Ultimately, SEEG is becoming more globally utilized for accurately capturing seizure foci, and in this analysis, allowed for successful localization in 95% of patients. These patients may go on to have open resective surgery but alternatively have the option of minimally invasive techniques for treatment of seizure foci, like LITT or radiofrequency ablation. Given similar outcomes, the selection of invasive monitoring techniques for a patient should thus be dictated by factors such as institutional availability of resources and expertise, clinical hypotheses of EZ localization, and may consider a combination of these methods for optimal results.

## Data Availability

The raw data supporting the conclusions of this article will be made available by the authors, without undue reservation.
